# Telescopic peristomes, hygroscopic movement and the spore release model of *Regmatodon declinatus* (Leskeaceae Bryophyta)

**DOI:** 10.1093/aobpla/plad073

**Published:** 2023-11-02

**Authors:** Yanzhi Wu, Zhihui Wang, Zhaohui Zhang

**Affiliations:** School of Life Sciences, Guizhou Normal University, Guiyang 550001, China; School of Life Sciences, Guizhou Normal University, Guiyang 550001, China; Key Laboratory for Information System of Mountainous Area and Protection of Ecological Environment of Guizhou Province, Guizhou Normal University, Guiyang 550001, China

**Keywords:** Mosses, peristome, *Regmatodon declinatus*, telescopic hygroscopic movement, the coupled release model of wind-water-capsule structural damage

## Abstract

Moss peristome hygroscopic movement plays an important role in protecting and controlling spore release. Recent studies on the peristome’s hygroscopic movement and spore release have focussed on mosses with ‘perfect’ peristomes, such as Brachytheciaceae, whereas the hygroscopic movement type and spore release pattern of ‘specialized’ peristomes, such as *Regmatodon declinatus*, are poorly understood. We investigated the relationship between the peristome’s hygroscopic movement and spore release in the ‘specialized’ peristome of *R. declinatus* by the measurement of peristome hygroscopic movement parameters and the hygroscopic movement spore release test. It was found that: (i) Exostomes (EX) are significantly shorter than endostomes (EN), triggering the hygroscopic movement of telescopic peristomes, in which the EX rapidly elongate while closing in on the EN, and the teeth rapidly converge. (ii) Spore release was minimal when peristome movement was triggered alone. The number of spores released when exposed to wind was 124 times greater than in the absence of wind. Dry capsules released seven times more spores than wet capsules. The study reveals that the hygroscopic movement of ‘telescopic’ peristomes of *R. declinatus* did not contribute significantly to spore release. More spores were released when wind and hygroscopic movement acted synergistically. Dry capsules released the maximum number of spores. It was also revealed that structural damage to capsules can facilitate complete spore release. Finally, we modelled the release of *R. declinatus* spores from initiation to complete release, namely the coupled release model of wind-water-capsule structural damage.

## Teaser

Because of their fine structure and imperceptible spore release, the relationship between moss peristome teeth (located at the top of the sporophyte) and spore release is not well understood. Using an original laboratory approach, we revealed the peristome movement and its effect on spore release, thus providing insights into the reproduction and dispersal of mosses. It was found that the subjects (*Regmatodon declinatus* ) mainly underwent telescopic movement after the structural degeneration of the teeth. When the movement is triggered, under ideal wind conditions, it does not better promote spore release. Some spores may not eventually spread over long distances, or they are not released until the sporophyte rots. Maybe the way moss spreads is more complicated than we thought. Finally, we propose a spore release pattern.

## Introduction

Peristomes are fascinating and important moss structures that can be divided into nematodontae and arthrodontae based on their developmental pattern (Mitten 1859; Fleischer 1900; Goebel 1906, 1930; Blomquist and Robertson 1941). Mature nematodontae peristomes are formed from whole cells. They move without any apparent change in spatial position. In contrast, arthrodontae peristomes are produced from cell wall remnants. The change in spatial position during movement is significant (Blomquist and Robertson 1941; Zhang *et al.* 2021). Macroscopically, the arthrodontae are divided into haplolepideae and diplolepideae (Taylor 1962). They are formed by the residual cell walls of different peristomial layers (William and Goffinet 2000). The diplolepideae are divided into two major groups based on morphological characteristics: ‘perfect’ and ‘specialized’ (Zanatta *et al.* 2018). The ‘perfect’ peristomes usually have wide EX and a high basal membrane in the EN, as in *Mitteniales* spp. and *Mnium* spp. (Shaw 1985). In contrast, ‘specialized’ peristomes are usually narrower, shorter or absent, with thinner EN and basal membranes (Hedenäs 2012), as in the Orthotrichaceae (Shaw 1986; Roads and Longton 2006; Fedosov *et al.* 2016).

The peristome regulates spore protection and release (Johnson 1931; Lazarenko 1957; Johansson *et al.* 2016). After operculum shedding, the peristome responds to changes in humidity. In dry conditions, it opens to form a passage for spore release (Budke *et al.* 2018). Goebel first identified an association between the hygroscopic movement of the peristome and spore release, which has since been confirmed in various mosses (Steinbrinck 1897; Pfaehler 1904; Ochyra and Garland 2016). Steinbrinck classified the hygroscopic movement of ‘perfect’ peristomes into three main categories: complete or partial inward bending of the EX, complete or partial outward bending of the EX and strong oscillatory movement of the EX during contracting and swelling (Steinbrinck 1897; Patterson 1953). Some of these three types of moss peristome movements occur in dry, wet and intermediate conditions (Lazarenko 1957; Zanatta *et al.* 2018; Bruggeman-Nannenga 2022). The recent studies on peristome movement and spore release (sporophyte vibration, wind speed and turbulence) in mosses have primarily focussed on ‘perfect’ peristome teeth, such as the nematodontae and arthrodontae of Brachytheciaceae (Lazarenko 1957; Ingold 1959; Kreulen 1972; Schnepf *et al.* 1978; Miles and Longton 1992; Stoneburner *et al.* 1992; Sundberg 2010; Johansson *et al.* 2014, 2016; Medina and Belen 2014; Lönnell *et al.* 2015; Ma *et al.* 2016; Bansal and Nath 2018; Gallenmüller *et al.* 2018; Zanatta *et al.* 2018; Zhang *et al.* 2021). The effect of ‘specialized’ peristome hygroscopic movement on spore release in arthrodontae is unknown. The peristomes of *R. declinatus* are ‘specialized’, and their hygroscopic movement differs from that previously reported.

The present study aimed to determine how the ‘specialized’ peristome teeth of *R. declinatus* undergo hygroscopic movements and to identify the spore release pattern in the moss. We analysed the peristome hygroscopic movement parameters, and conducted hygroscopic movement spore release experiments and indoor simulated wind-blowing experiments to investigate the mechanism of spore release in *R. declinatus* and to explain the function of the degraded peristome. This study advances our understanding of moss dispersal and reproduction strategies.

## Materials and Methods

### Field sampling

Specimens were collected from the trunk of a Chinese sweetgum (*Liquidambar formosana*) growing in Baisang Village, Maojian Town, Duyun City, Guizhou Province, China. Samples were collected by scraping off mature capsule-bearing plants with a small spatula from 30 to 176 cm above the ground, along with the bark, and transported to the laboratory in envelope bags. The specimens were stored in the Bryophyte Herbarium at the College of Life Sciences, Guizhou Normal University, China (No. 2021080701).

### Research species

**Figure 1.**
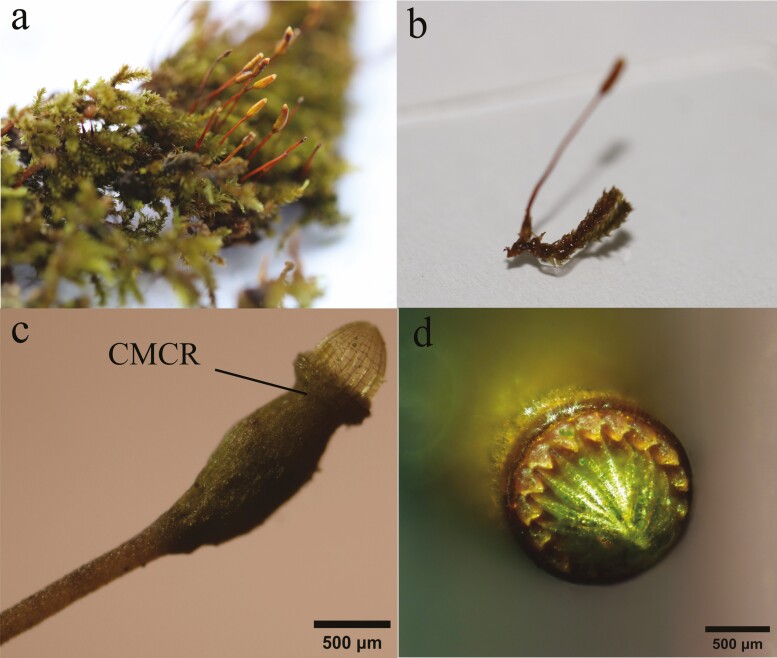
Research species; (a) *R. declinatus* plant; (b) Part of the plant body; (c) Lateral view of the capsule; The capsule mouth contraction region; (d) Top view of the peristomes.

*Regmatodon declinatus* is a member of the *Regmatodon*, the Leskeaceae and the Hypnobryales. The capsule of *R. declinatus* grows sideways and upright (Fig. 1a, b). The peristomes are a ‘specialized’ type of diplolepideae, with the EX shorter than the EN, which are yellowish, hyaline and with a low basal membrane. The species grows on tree trunks or rocks (Wu 2002). The capsules mature in summer, followed by the shedding of the calyptra and operculum, releasing spores from the upper capsule opening.

### Measurement of peristome hygroscopic movement parameters

**Figure 2.**
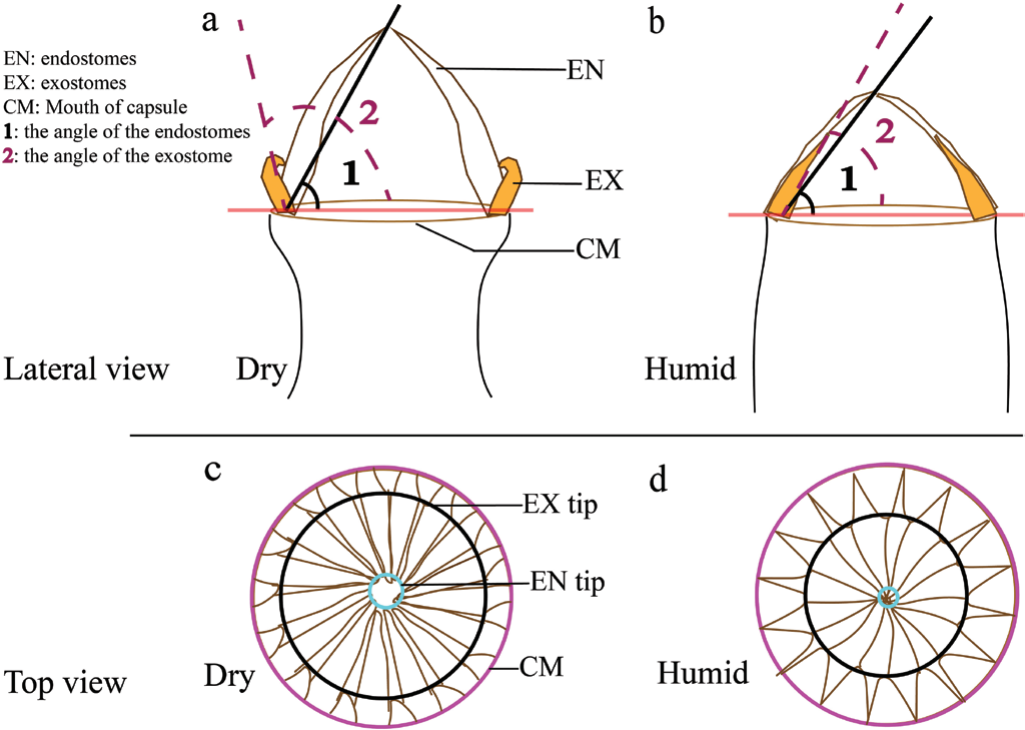
Measurement of the opening angle and circumference of peristomes; (a) Peristome angle measurement in the dry state; (b) The peristomes angle measurement in the humid state; (c) Dry state circumference measurement (The ‘magenta circle’ is the circumference of the CM; ‘The black circle’ is the circumference of the EX tip; ‘The cyan circle’ is the circumference of the EN tip); (d) Wet state circumference measurement.

Ten capsules of similar size were selected for drying (natural air drying for 12 h). A microscope (OLYMPUS CX41) was used to measure capsule width, CMCR width (Fig. 1c), peristome opening angle (lateral view), peristome cusp circumference and capsule opening circumference (top view) (Fig. 2). All these structural parameters were measured again in the wet condition. Peristome length was measured by dissecting it with an HWG-1 binocular dissecting microscope and then filming it. All measurements were repeated and averaged.

### Spore counting

Five spore-filled capsules were selected, and the total number of spores was counted. A single capsule was dissected in 500 µL of pure water in a petri dish, the spores in the capsule were rinsed out with pure water, mixed well and a 1000 µL spore suspension was made, from which a 10 µL sample was taken for counting by placing it on a counting slide. Spores in each capsule were counted five times, and the average value was calculated.

### Hygroscopic movement and spore release test for *R. declinatus* peristomes

**Figure 3.**
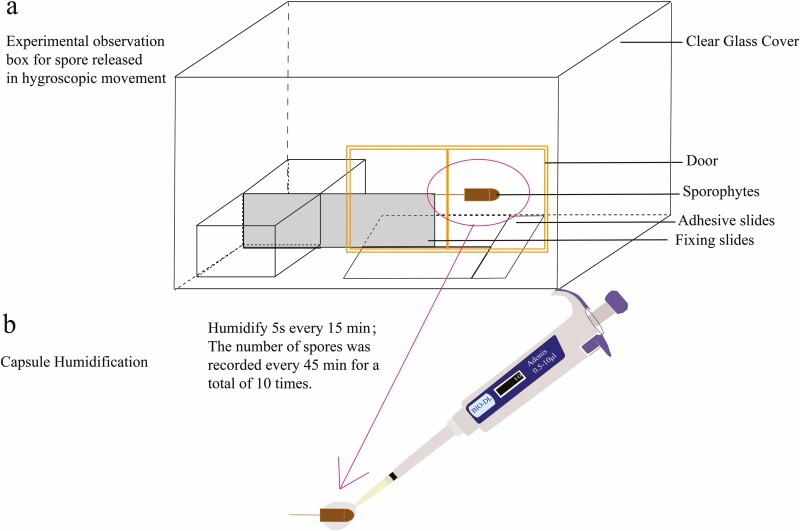
Experimental set up for hygroscopic movement spore release test; (a) Test observation box; (b) the method of capsule humidification.

Six similar sporophytes were collected and randomly assigned to experimental and control groups. In the experimental group, the capsules were submerged in water to trigger hygroscopic movement (Fig. 3b), with humidification for 5 s every 15 min, and the number of spores released was recorded after three periods of humidification (45 min), on a total of 10 occasions (450 min). The experiment was repeated three times for each capsule. The experiments were performed in a sealed box (Fig. 3a).

Indoor simulated wind-blowing experiments were conducted (Zhang *et al.* 2021). Six similar sporophytes were randomly assigned to experimental and control groups. The investigation included comparative wind, no wind experiments and controlled hygroscopic movement under constant wind conditions. The wind was blowing at 7.5 m/s at a distance of 10 cm in the experimental group. The number of spores released was counted when the wind blew for 5 s at 2 min intervals, with a total of 60 counts being made.

### Data analysis

All data were analysed using SPSS (R27.0.1.0). After analysis of normality, an independent samples *T*-test, Friedman test or Wilcoxon signed rank test was conducted to determine the significance of differences in the structure and spore release data. Images were drawn using Adobe Illustrator 2020 (24.0.1) and Origin 2021 (9.8.0.200).

## Results

### Hygroscopic movement of *R. declinatus* peristomes

**Figure 4.**
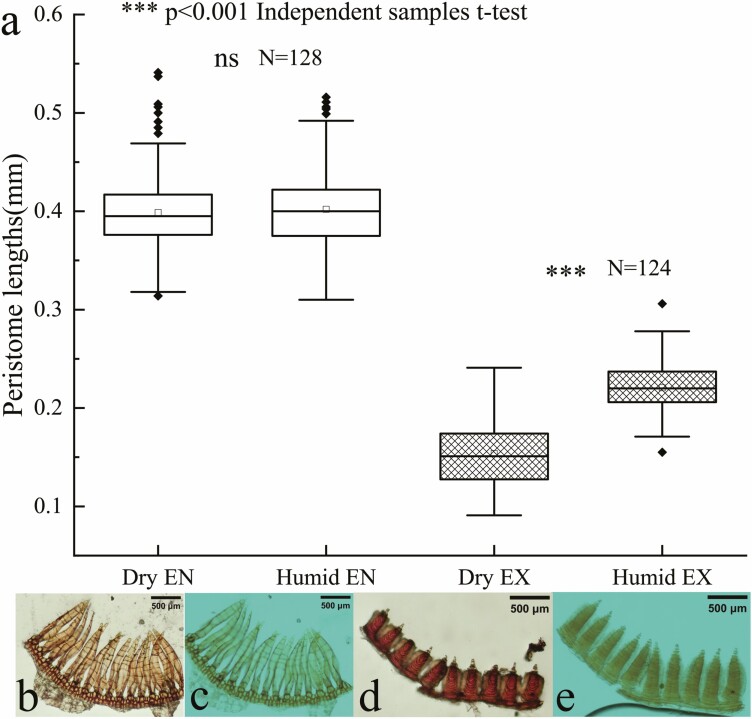
Variation in *R. declinatus* peristome length; (a) Length change of EN and EX; (b) Dry EN; (c) Humid EN; (d) Dry EX; (e) Humid EX. ns. Indicates that the change in internal peristome length in dry and wet states was not statistically significant.

The measurement of peristome hygroscopic movement parameters indicated that the exostomes (EX) were shorter than the endostomes (EN). In dry conditions, the length of EX was 0.15 mm; the EX elongated rapidly after triggering hygroscopic movement, with a length of 0.22 mm; EX was 1.44 times longer in wet conditions than in dry (Fig. 4a, d and e). The length of EN in dry conditions was 0.40 mm. The length after triggering the hygroscopic movement was 0.40 mm, with no significant difference in length between the dry and wet conditions (Fig. 4a-c).

**Figure 5.**
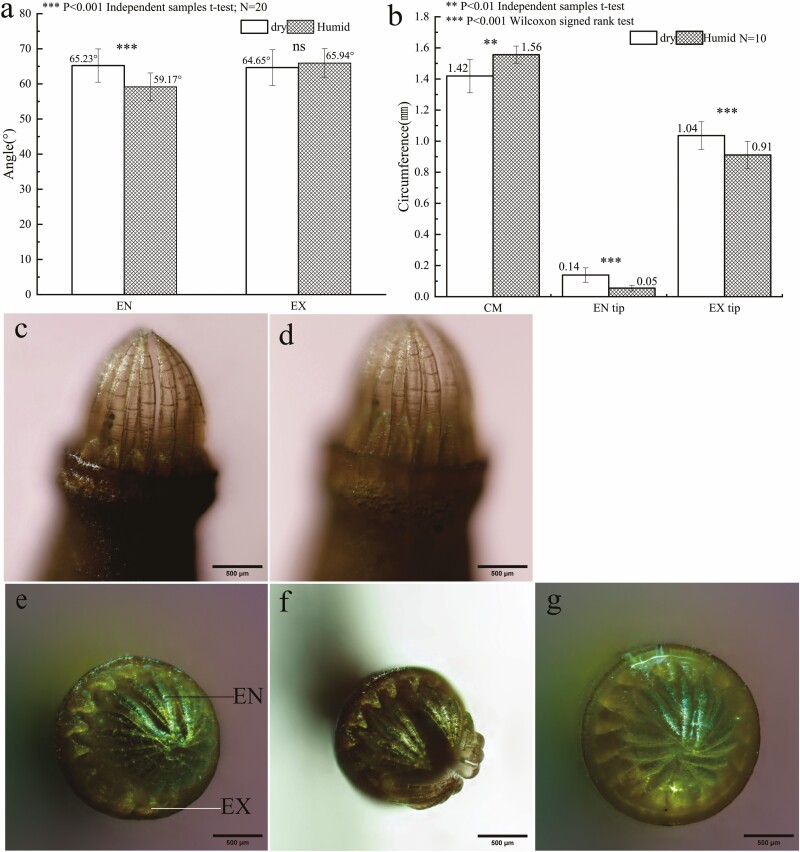
Analysis of peristome hygroscopic movement in dry and wet states; (a) Changes in tension angle of EN and EX; (b) Changes in the perimeter of CM, EN tip and EX tip circumference in dry and wet states; (c) Dry EN and EX; (d) Wet EN and EX; (e) Uniformly dispersed ENs in a dry state; (f) Clustered and dispersed EN in a dry state; (g) Closed EN in the wet state. ns indicates that the difference in the tension angle of EX in dry and wet conditions was not statistically significant.

When observed laterally, it was found that when the hygroscopic movement was triggered, the tip of the EX rapidly straightened and then attached to the EN without vibratory motion in this process. The angle shifted from 65° to 66° without significant change. Meanwhile, the EN angle was reduced from 65° to 59°, with significant differences in the variation of the tension angle (Fig. 5a, c and d).

In the top view, uniform and clustered dispersion of endostome tips (EN tip) was observed during drying (Fig. 5e, f). After triggering the hygroscopic movement, the EN tip gathered inward to form a significantly smaller circle, whose circumference reduced from 0.14 mm to 0.06 mm. The exostome tip (EX tip) curved slightly inward to form a larger circle during drying. However, after triggering the movement, the EX tip straightened and fitted tightly with the EN to seal the tooth gap, resulting in a smaller circle around the tip, reducing the circumference from 1.04 mm to 0.91 mm (Fig. 5b, e and g). Simultaneously, there were expansion movements in the capsule mouth (CM), changing the circumference from 1.42 mm to 1.56 mm (Fig. 5b).

### The effect of *R. declinatus* peristome hygroscopic movement on spore release

**Figure 6.**
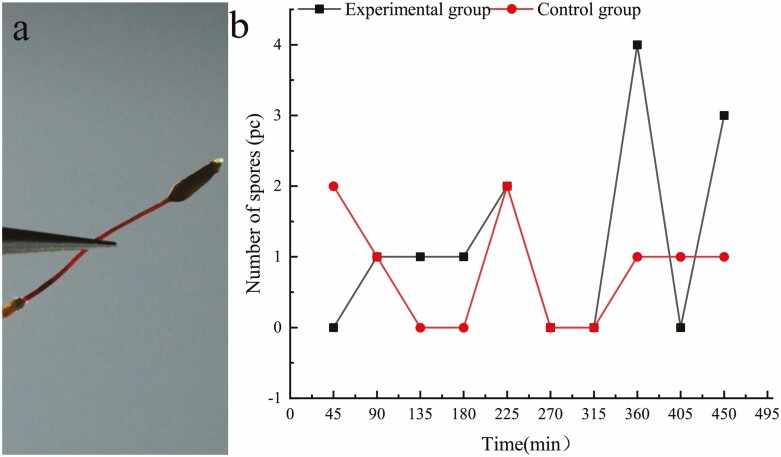
Effect of peristome hygroscopic movement on spore release; (a) Spore-filled capsule; (b) Trends in spore release with (experimental group) and without (control group) triggering peristome hygroscopic movement.

The number of spores released in the hygroscopic movement spore release experiments was minimal when only peristome movement was triggered. Spore release trends in the experimental and control groups varied from 0 to 4 spores (Fig. 6a, b). The average number of spores (*M*) in a single capsule was 6.50 × 10^4^. Simultaneously, there was no significant difference in spore release between those that triggered movement and those that did not, and their spore release numbers and rates were low (Table 1).

**Table 1:** Statistics on the number of spores in a single capsule and the number of spores released.

	Total spore (*M*)	Spore release (*m*)	Spore release rate (*R*)
Experimental group	6.50 × 10^4^ ± 2212.402	335 a	0.52 %
Control group	6.50 × 10^4^ ± 2212.402	221 a	0.34 %

Note: *R* = *m*/*M* × 100 %; experimental group, control group (*M*, *N* = 25; m, *N* = 90). The same letter indicates that the difference in the number of spores released between E and C was not significant.

### Effects of wind and *R. declinatus* peristome hygroscopic movement on the release of spores

**Figure 7.**
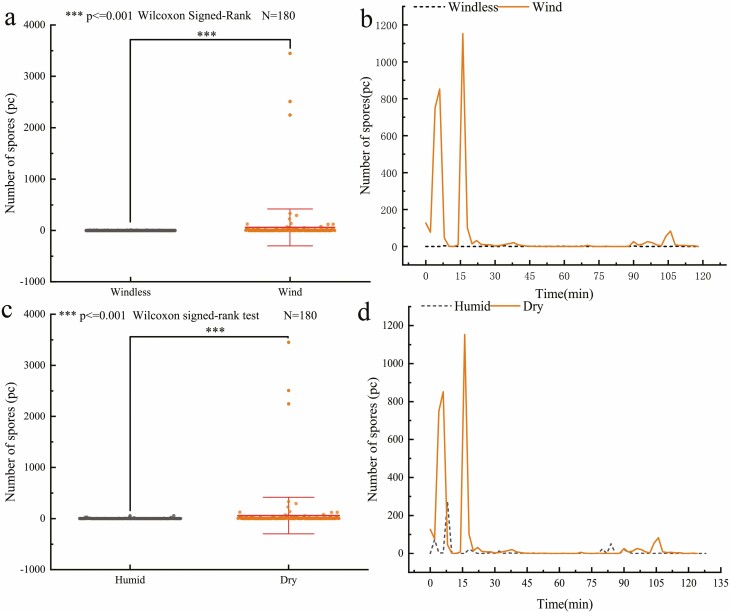
Effect of wind and peristome hygroscopic movement on spore release; (a) Differences in the number of spores released between windy and windless conditions; (b) The spore release trends in windy and windless conditions; (c) Differences in the number of spores released between dry and humid states; (d) The spore release trends between dry and humid states (humidification treatment: 0 min for first capsule submergence treatment).

In the indoor simulated wind-blowing experiments, the number of spores released was 10 638 in the presence of wind, but there were no hygroscopic movements. The trend in number of spores released fluctuated at first, then decreased, and finally approached zero. When there was no wind, the average number of spores released was 86. Spores were released randomly and sporadically. There was no clear trend. The total number of spores released in the wind was 124 times higher than in the absence of wind (Fig. 7a, b).

Under constant wind-blast conditions, spore release was higher and more concentrated at the beginning of a drying wind-blast (0–38 min), followed by low-level sporadic release. In the late stages (80–118 min), spores were again released under wind vibration, with an insignificant change in the release trend. Wetting the capsule triggered the hygroscopic movement, and the average number of spores released was 1583. The release trends were similar to those in the dry state, but there was a low release level. When the capsule was wet (0 min, 15 min, etc.), the peristome closed, and no spores were released. Spore release remained low after the capsule resumed drying. The number of spores released in the dry condition was seven times higher than that released in the wet state (Fig. 7c, d).

## Discussion

### The telescopic hygroscopic movement of ‘specialized’ peristomes of *R. declinatus* and its regulation of spore release

The study showed that the hygroscopic movement occurred when the EX significantly elongated and adhered to the EN, while causing the EN to converge. During the drying process of the capsule from wetting to drying, the EX tip first curved inwards slightly before the entire tooth plate contracted rapidly (Figs 4 and 5). This type of hygroscopic movement differs from previous descriptions (Steinbrinck 1897). Therefore, we call this type of hygroscopic movement ‘telescopic’ hygroscopic movement, in which the length of the EX changes significantly. However, the tension is not obvious, and there is no oscillation during the motion.

Moss peristomes differ in structure and function depending on their environment (Bruggeman-Nannenga 2022). *R. declinatus* often grows on tree trunks or rocky surfaces (Wu 2002). The morphological specialization of their peristomes is due to their adaptability to arid habitats, as evident by the shortening or narrowing of their peristomes, resulting in the degradation or loss of some of their functions (Vitt 1981; Huttunen *et al.* 2012). It has been demonstrated that the ‘specialized’ peristomes of *R. declinatus* form small spore release channels during drying, leading to a minimal number of spores being released when only telescopic hygroscopic movements occur (Fig. 6, Table 1). Furthermore, spores located at the mouth may be dispersed under the influence of gravity (Gregory 1945). Further research is required to investigate the physical mechanisms of *R. declinatus* ‘telescopic’ hygroscopic movement and that of peristomes in other species.

When the *R. declinatus* peristomes are not damaged and are capable of normal hygroscopic movement, the spores are released by the synergistic action of water and wind.

Numerous studies have demonstrated that arthrodontae spore release is wind-driven (Gregory 1945; Johansson *et al.* 2014, 2016), as further verified by the present study. However, it was observed that, when peristomes are not damaged, they are capable of normal hygroscopic movement; Spores were not released in large quantities continuously under dry capsule conditions but tended to be released at a low level after a concentrated explosive release (Fig. 7a, b). It was also evident that when the capsule became dry, the combined action of hygroscopic peristome movement and wind failed to restore a concentrated mass release of spores, and the overall trend in release was suppressed (Fig. 7c, d). Moss spore release may be related to more than peristome movement (Merced and Renzaglia 2013; Chater *et al.* 2016; Caine *et al.* 2020). Low release levels are also present in *Polytrichum commune* (Zhang *et al.* 2021). This is related to the contraction and expansion of the capsule (Supporting Information—Fig. S1a–c). This suggests that the hygroscopic movement of the capsule and peristome regulates *R. declinatus* spore release.

Previous studies have shown that the presence of longitudinal folds (8–16) in the capsule of plants in the Orthotrichaceae reduces the volume of the capsule as it dries, extruding or pushing the spores towards the mouth, where they are released (Paton and Pearce 1957; Vitt 1981; William and Goffinet 2000). The open state of the peristome teeth of *R. declinatus* during drying and the contraction of the capsules differs in the presence of the CMCR (Fig. 1c). It is because of the uneven absorption of water by the wall cells that the contraction of this part is more significant (Vitt 1981). It is thought that the CMCR prevents the spores at the CM from ‘refluxing’ during drying and controls the spores’ upward movement by forming the CMCR in the dry environment, preventing the spores at the mouth from being replenished and released.

Disruption of the capsule structure after the ageing of the *R. declinatus* capsule is required to facilitate complete spore release.

**Figure 8.**
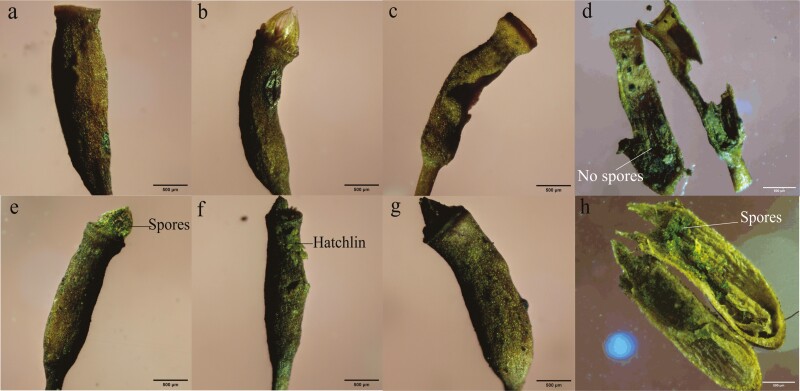
*Regmatodon declinatus* capsules in different states after ageing; (a) The peristomes have been shed; (b) The peristomes are not shed, and the capsule was dehiscence inconspicuous; (c) The peristomes are shed, and the capsule was dehiscence obvious; (d) The capsule ‘c’ without remaining spores after dissection (dry); (e) The peristomes were not shed, and the capsule was not cracked; (f) The peristome is clearly collapsed, and a new plant (hatchling) is growing on the outer wall of the capsule; (g) The peristome in a sticky state, and the capsule is not cracked; (h) The capsule ‘g’ with spores still inside after dissection (dry).

In the later stages of the ideal indoor wind-blown experiments, spore release under all experimental conditions eventually converged to a low level of release (Fig. 7b and d, and Supporting Information—Fig. S1e), prompting further thoughts on whether spores can be fully released in the wind and under hygroscopic peristome movement alone. We then observed the capsule after ageing in the field under natural conditions and identified that it had suffered varying degrees of damage (Fig. 8a-c). There were no residual spores when the peristomes were completely detached and the capsules split (Fig. 8c, d); otherwise, there were residual spores inside the ageing capsule (Fig. 8e, g and h) or the spores germinated directly on the surface (Fig. 8f).

Therefore, we suggest that the *R. declinatus* spore release process, from the beginning to the end, combines telescopic and hygroscopic peristome movement and capsule damage in different stages (Gao *et al.* 2000). When there are numerous spores, and hygroscopic movement of telescopic peristomes is normal, the peristome teeth and the capsule jug collectively control the continuous release of spores. When the number of spores remaining is very low, the peristomes may act as a barrier to spore release (Lazarenko 1957). Damage to the capsule structure will result in complete spore release (Fig. 8a-c). Otherwise, spores are not fully released. Therefore, spores may germinate directly in the capsule to improve germination and reproduction efficiency within the capsule (Fig. 8e, f) or may wait until it has rotted and fallen off (Steinbrinck 1897; Caparros *et al.* 2011).

**Figure 9.**
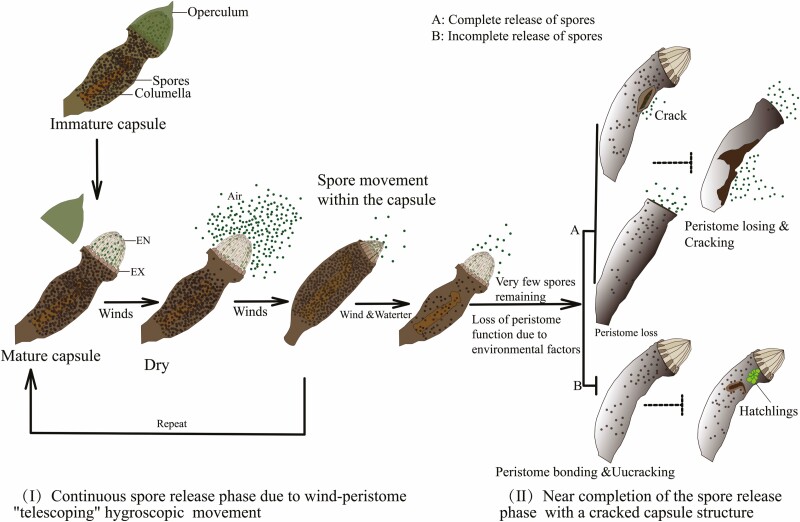
The coupled release model of wind-water-capsule structural damage for *R. declinatus* (Dashed lines indicate significance, requiring further confirmation).

In summary, we modelled the mechanism of continuous spore release and complete spore release following capsule structure damage caused by the hygroscopic movement of the telescopic peristomes of *R. declinatus* (Fig. 9). The relationship between capsule structure damage and its own or environmental factors requires further investigation.

## Conclusion

The findings reveal that the *R. declinatus* peristomes are specialized, with exostomes (EX) shorter than the endostomes (EN), resulting in telescopic hygroscopic movement. This movement regulates spore release. *R. declinatus* spores are better suited for release in dry environments when the peristome structure is not damaged, and hygroscopic movement is possible. The spore release process under the combination of water and wind is continuous. It is also controlled by the hygroscopic movement of the peristome and the capsule. When the number of spores in the capsule is very low, the peristome becomes an obstacle to spore release. The destruction of sporophyte structure and spore germination in the capsule promoted the spread and germination efficiency of spores to a certain extent. Therefore, *R. declinatus* spore release from start to end results from the combined effect of wind-water-spore structure destruction.

## Supporting Information

The following additional information is available in the online version of this article—

Figure S1. The hygroscopic movement and structural changes of capsules; (a) The capsule in the dry state; (b) The capsule in the humid state; (c) Variation in capsule and CMCR width; (d) The number of spores released varied under different peristome treatments (intact (P) or removed (RP)), dry (D) and humid (H) conditions; (e) Trends in spore release from dry and wet capsules with peristomes removed.

plad073_suppl_Supplementary_Figures_S1

## Data Availability

The data that support the findings of this study are incorporated into the article and its Supporting Information. Further inquiries can be directed to the corresponding author.
