# Supplementary material for: Telescopic peristomes, hygroscopic movement and the spore release model of Regmatodon declinatus (Leskeaceae Bryophyta)
Source: AoB Plants. 2023 Nov 2;15(6):plad073. doi: 10.1093/aobpla/plad073 (PMC10656297; doi:10.1093/aobpla/plad073)
Supplement: plad073_suppl_Supplementary_Figures_S1 [file plad073_suppl_supplementary_figures_s1.docx]

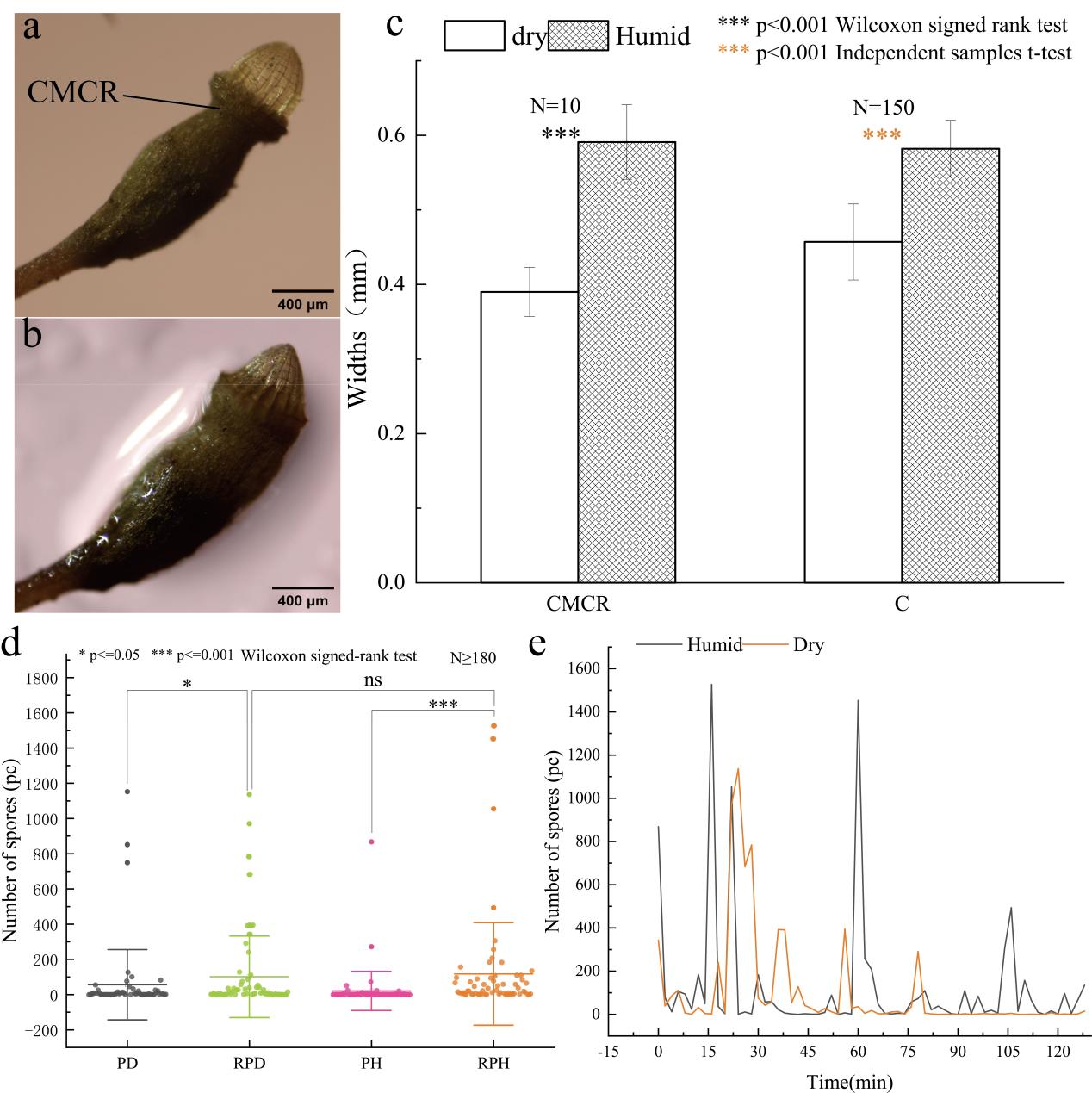


Figure S1 The hygroscopic movement and structural changes of capsules; a. The capsule in the dry state; b. The capsule in the humid state; c. Variation in capsule and CMCR width; d. The number of spores released varied under different peristome treatments (intact (P) or removed (RP)), dry (D) and humid (H) conditions; e. Trends in spore release from dry and wet capsules with peristomes removed.
